# Human Dorsal Root Ganglia Neuronal Cell Line to Study Nociceptive Signaling: A New Pipeline for Pain Therapy

**DOI:** 10.1096/fj.202503698R

**Published:** 2026-02-03

**Authors:** Sara A. Dochnal, Yixing Du, Daniella Bandari, Kaue Franco Malange, Jack Bryant, Julia Borges Paes Lemes, Abby Whitford, Anna R. Cliffe, Prashant Mali, Kim Dore, Yury I. Miller, Tony L. Yaksh

**Affiliations:** ^1^ Department of Anesthesiology University of California San Diego San Diego California USA; ^2^ Department of Neurosciences, Center for Neural Circuits and Behavior University of California San Diego San Diego California USA; ^3^ Department of Bioengineering University of California San Diego San Diego California USA; ^4^ Department of Microbiology University of Virginia Charlottesville Virginia USA; ^5^ Department of Medicine University of California San Diego San Diego California USA

**Keywords:** afferent signaling, dorsal root ganglia, HD10.6, human model, microfluidics, nociceptor

## Abstract

Nociceptive afferent neurons within the dorsal root ganglion (DRG) detect and relay painful peripheral stimuli, and the malfunctioning of this process leads to sustained pain states. Animal model studies have been invaluable for demonstrating the importance of the DRG nociceptor in pain sensation and the development of related analgesic targets. However, a human in vitro model of nociception is essential to confirming the relevance of preclinical findings for therapeutic drug development. We characterized the nociceptive properties of differentiated cells from the human DRG‐derived immortalized cell line HD10.6 and developed their use into an in vitro model of human nociceptive signaling and therapy. Within differentiated HD10.6 cells, we confirmed the abundance and function of machinery linked with pain sensation, including key ion channels (TRPV1, NaV1.7) and afferent peptides (CGRP, Substance P), by immunofluorescence and calcium influx assays. Through whole‐cell patch clamp, including current clamp and voltage clamp, we recorded the baseline electrophysiological parameters of differentiated HD10.6 cells. We further found that differentiated HD10.6 cells express the mu opioid receptor 1 protein, and that mu agonist DAMGO blocks depolarization‐evoked calcium influx in a naloxone‐reversible fashion. Importantly, excitation and peripheral sensitization were induced within HD10.6 cells in response to an inflammatory cocktail, mirroring nociceptors in a pain state during and after tissue damage or inflammation. HD10.6 cells were also cultured into dual‐chambered microfluidic devices to mirror the physiological anatomy of the nociceptor. Within this system, genetic therapy adeno‐associated‐virus was successfully taken up by the peripheral terminals and transported to the soma.

## Graphical abstract

The nociceptive properties of differentiated human HD10.6 cells were characterized by multiple approaches (A). Peripheral sensitization was induced within HD10.6 cells in response to an inflammatory cocktail (B), modeling nociceptors in a chronic pain state. HD10.6 cells within dual‐chambered microfluidic devices underwent peripheral uptake and transport of genetic therapy adeno‐associated virus (C). We developed the use of HD10.6 cells into an in vitro model of human nociception for the investigation of molecular mechanisms of nociceptive signaling and its therapy.

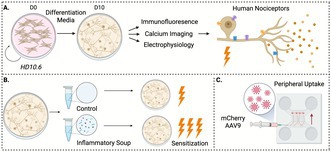

## 1 Introduction

Following peripheral tissue injury and inflammation, afferent signaling has been shown to underlie many aspects of the consequential pain phenotype. Decoding the physiology of afferent signaling through experimental models is essential to the creation of therapeutics that selectively regulate the pain state they subserve. While many physiological nociceptive pathways are conserved [1, 2], there are nonetheless essential anatomical, cellular, molecular, transcriptional, and electrophysiological differences between DRG neurons from animal models and humans (for review, we recommend [1, 3]). Species‐specific transcriptomic differences in expression patterns of key genes implicated in pain signaling have been recently highlighted by a series of publications that have taken high resolution sequencing approaches to human DRGs [4, 5, 6, 7, 8]. In view of the large‐scale failure of clinical trials based on animal models [9], the success of new drugs to treat pain in the clinic will likely require complementary studies of human cells and tissues.

In 1999, Raymon et al. created a human‐derived DRG neuronal cell line entitled “HD10.6” [10]. Cells from DRGs were isolated and immortalized with a tetracycline‐inducible c‐myc retroviral vector, resulting in a passable clone of neural precursors. Following the addition of tetracycline to suppress c‐myc expression and several growth factors to facilitate differentiation, the precursors develop into cells with sensory neuron properties. Differentiated HD10.6 cells transmit action potentials [10] and demonstrate immunoreactivity for several filaments, receptors, and transcription factors characteristic of DRG sensory neurons [10, 11, 12]. Therefore, HD10.6 cells have the profound potential to provide species‐specific insight into DRG neuron physiology. However, the use of this cell line has been limited since its introduction. HD10.6 cells require further characterization at the RNA, protein, and functional level to evaluate their suitability as a nociceptive system for studying peripheral pain sensation and further optimization to maximize their utility to ultimately be used as a drug pipeline assay.

In this manuscript, we characterized HD10.6 cells as *de facto* human nociceptors. We highlighted the abundance and behavior of characteristic nociceptive ion channels, excitatory/inhibitory receptors, and afferent peptides, including Transient Receptor Potential Vanilloid 1 (TRPV1), voltage‐gated sodium channel NaV1.7, calcitonin gene‐related peptide (CGRP), Substance P, neurokinin‐1 receptor (NK1R), and MOR. Using whole‐cell patch clamp, we defined electrophysiological parameters of the differentiated HD10.6 cells for later comparison to the parameters of human patient DRGs and nociceptor‐like induced pluripotent stem cells. Importantly, we induced and primed excitability in HD10.6 cells using an inflammatory cocktail to mirror nociceptors in a pain state during and after tissue damage or inflammation. HD10.6 cells were also cultured into dual‐chambered microfluidic devices, in which we can isolate the soma and axon terminals of the nociceptor and mirror the nociceptor's biological anatomy. HD10.6 cells displayed successful AAV infection following administration to the peripheral terminal, mirroring the peripheral delivery of pain therapeutics. Altogether, we have used HD10.6 cells to effectively model nociceptor physiology and therapeutic intervention and anticipate that these data will advance our understanding of human molecular pain mechanisms and therapy.

## 2 Materials and Methods

A list of reagents and their respective catalog numbers and concentrations used is presented in Table 1.

**TABLE 1:** Summary of reagents' sources and working concentrations.

Reagent	Catalog number	Working concentration
Capsaicin	Sigma M2028	10 μM
OD1	Smartox Biotechnology	0.5 μM
KCl	Sigma P5405	10 mM, 50 mM
Forskolin	Selleckchem S2449	25 μM
DAMGO	Tocris 1171	1 μM
Naloxone hydrochloride dihydrate	Sigma N7758	10 μM
Bradykinin	MedChemExpress HY‐P0206	1 μM
Prostaglandin E_2_	Tocris 2296	10 μM
Serotonin hydrochloride	MedChemExpress 153‐98‐0	10 μM
ATP	Sigma A7699	15 μM
Histamine	Sigma H7125	10 μM

### 2.1 HD10.6 Cell Culture

All cells were incubated at 37°C and 5% CO_2_. Proliferation and differentiation protocols were guided by previous publications [10, 11]. Immortalized human dorsal root sensory ganglion cells (HD10.6 cells, RRID:CVCL_WG82 [10], male, passage number 20–24) were passaged in proliferation media on Nunc flasks (153732; Thermo Fisher Scientific) coated for 3 h with 17 μg/μL fibronectin (33016‐015; Thermo Fisher Scientific) in 1X PBS. Proliferation media consisted of Advanced Dulbecco's modified Eagle medium (12634010; Gibco Life Technology) supplemented with glutaMAX (35050061; Gibco), Prime‐XV IS21 (91142; Fujifilm), and 10 ng/mL prostaglandin E1 (P5515; Sigma). Fresh 0.5 ng/mL fibroblast growth factor‐basic (bFGF) (03‐0002; Stemgent) was added prior to media change. HD10.6 cells tested negative to presence of mycoplasma prior to any experimentation.

For differentiated HD10.6 cells, glass coverslips (Fisher Scientific) were prepared in advance to enhance adherence. Coverslips were coated with 10 μg/mL Poly L‐ornithine (P4957; Sigma) in borate buffer (BB‐66; Boston Bioproducts) overnight at 37°C and washed three times with sterile water (W3500; Millipore Sigma) the following morning. The coverslips were then coated with 1 μg/mL fibronectin in sterile water overnight at 37°C, washed three times with sterile water the next morning, and left to dry within a sterile tissue culture hood for 24–72 h. Cells were seeded on day 0 (D0) at a density of ~16 000 cells/cm^2^ in proliferation media on coated coverslips. Media was changed to complete differentiation media 24 h after plating (D1), with half‐volume medium changes performed every 2 days afterwards. Complete differentiation media consisted of Neurobasal ‐phenol red ‐L‐Glutamine (C12348‐017; Gibco‐Life Tech) supplemented with glutaMAX (35050061; Gibco), Prime‐XV IS21 (91142; Fujifilm), 50 ng/mL 2.5S nerve growth factor (NGF) (N‐100; Alomone Labs), 25 ng/mL of ciliary neurotrophic factor (CNTF) (450‐13; PeproTech), 25 ng/mL glial cell‐derived neurotrophic factor (GDNF) (G‐240; Alomone Labs), and 25 ng/mL neurotrophin‐3 (NT‐3) (450‐03; PeproTech). Fresh 1 μg/mL doxycycline (T‐7660; Sigma) was added prior to media change.

### 2.2 Immunofluorescence

HD10.6 cells on coated 12 mm circle glass coverslips were washed twice with 1X PBS and fixed for 10 min on ice with 4% paraformaldehyde (PFA). Cells were blocked using 2% fetal bovine serum (FBS), 0.2% Triton X‐100, and 2% serum from the appropriate host species (donkey or goat) of secondaries for 1 h at room temperature. Cells were incubated with primary antibody diluted in blocking buffer for 2 h at room temperature. Following three 1X PBS washes, coverslips were incubated with secondary antibody diluted in blocking buffer for 1 h at room temperature, covered from light. Following three 1X PBS washes, coverslips with cells were mounted on slides with ProlongGold containing DAPI (Thermo Fisher) and imaged using a Leica SP8 confocal microscope. Images were analyzed using imagej, RRID:SCR_003070. Image acquisition and analysis was blinded. A list of antibodies and their respective catalog numbers and working dilutions is presented in Table 2.

**TABLE 2:** Summary of antibodies' sources and working dilutions.

Antibody	Catalog number	Working dilution
NF200	Millipore AB5539	1:1000
β3‐Tubulin	Millipore AB9354	1:100
TRPV1	Novus Biologicals NBP1‐97417	1:200
NaV1.7	Alomone Labs ASC‐008	1:100
CGRP	Sigma Aldrich C8198	1:100
Substance P	Merck Millipore A1566	1:80
NK1R	Merck Millipore AB5060	1:80
MOR1	Abcam ab10275	1:80
ATTO488‐ProTx‐II	Smartox	1:200
mCherry	Abcam ab167453	1:500

### 2.3 Calcium Imaging

All calcium imaging experiments were performed on differentiated HD10.6 cells plated on coated 15 mm glass coverslips. HD10.6 cell cultures were loaded with the Ca^2+^ indicator Fluo‐4 AM (5 μM; F23917; Invitrogen) with PowerLoad Concentrate (P10020; Thermo Fisher) in differentiation media without doxycycline for 1 h at room temperature. Coverslips were placed in a laminar flow perfusion chamber (Warner Instrument Corp., UK) and continuously perfused with extracellular solution (in mM: NaCl 160; KCl 2.5; CaCl2 1; MgCl2 2; HEPES 10; glucose 10; pH 7.4). The perfusion system consisted of up to four individual compartments filled with treatment solutions, each one connected to a separate cannula that merged to one output cannula. The latter was inserted in the laminar flow perfusion chamber containing a glass coverslip with the HD10.6 cells. The flow was constant and the HD10.6 cells were always covered in solution. Each treatment valve was opened manually by the experimenter. A suction pump connected to the outflow side of the chamber maintained a continuous perfusion. HD10.6 cells were recorded in an inverted Leica TCS SP5 confocal microscope. One field of view per coverslip was assessed. Cells were exposed to buffer, then stimulated with drug diluted in buffer with times of exposure as specified within figure legends. All cultures were stimulated with 50 mM KCl for 5 s at the end of experiments to confirm cellular viability. Neuronal Ca^2+^ responses were analyzed in 3–5 dishes per biological replicate by selecting individual cells as ROIs (Region of Interest) and calculating mean gray value variations on each individual cell using LAS AF version 2.7.3.9723 software. Data are presented as ΔF/F0, where F0 is the baseline, and the effect is quantified as maximal ΔF/F0. Randomization was applied during treatment. The experimenter was blinded to conditions during analysis.

### 2.4 Electrophysiological Recordings

Whole‐cell patch clamp was performed on intact, differentiated HD10.6 cells plated on coated 15 mm circle glass coverslips. Coverslips were transferred into the recording chamber with a continuous flow of extracellular solution containing 150 mM NaCl, 4.0 mM KCl, 2.0 mM CaCl_2_, 2.0 mM MgCl_2_, 10 mM glucose, and 10 mM HEPES (pH 7.4). The extracellular solution was perfused at a rate of 1.5–2.0 mL/min and heated to 30°C–32°C with a temperature controller (TC‐324B; Warner Instrument). The cells were allowed to rest in the chamber for 5–10 min before all recordings. HD10.6 cells were identified under the microscope of the recording rig by their small, oval‐shaped cell body. Borosilicate glass pipettes (outer diameter: 1.5 mm, Warner, Hamden, CT) were pulled from a micropipette puller (Model P‐97; Sutter). The recording electrodes had a resistance of 2–5 MΩ when filled with an internal solution containing 130 mM potassium gluconate, 1.0 mM MgCl_2_, 1.0 mM CaCl_2_, 10 mM HEPES, 4 mM MgATP, 0.3 mM Na_2_GTP, and 5.0 mM EGTA at pH 7.2. A MultiClamp 700B amplifier, an Axon Digidata 1550B, and Clampex 11 software (Molecular Devices, San Jose, CA, USA) were used for data acquisition, digitized at 2–10 kHz, and filtered at 2 kHz. The liquid junction potential was compensated for prior to forming the cell‐attached mode for all recordings. A minimum of 2 GΩ seal resistance was required before rupturing the membrane for whole‐cell configuration. Membrane test in Clampex software was used to read the membrane capacitance (Cm), membrane resistance (Rm), and access resistance (Ra) immediately after whole‐cell configuration. The cells were recorded in current clamp mode first to acquire resting membrane potential (resting Vm) without holding current, rheobase (10 ms duration of minimal current injection from 0 pA with 10 pA increment until the first action potential appears), and excitability (500 ms duration of −50 to +500 pA current injection steps with 50 pA increment). Then the cells were held at −70 mV in voltage clamp. When the holding current stabilized, a voltage step protocol was applied to record the Na^+^ currents and K^+^ currents. Voltage steps were from −200 to +40 mV with 10 mV increment and 25 ms duration, and with the baseline holding at −80 mV. Only the cells with Ra < = Rm × 10% and less than 20% fluctuation of Ra were included for voltage clamp recordings. Multiple cells were recorded from each coverslip; and each coverslip was kept in the recording chamber for less than 2 h to ensure that the cells were recorded in a healthy state. Randomization was applied during treatment.

All the data were analyzed in software ClampFit (Molecular Devices). For excitability analysis, “Threshold search” function in ClampFit was used; the depolarization peaks with overshoot above 0 mV were identified as action potentials, and only action potentials occurring during the current injection period were included for quantification of induced action potentials. For the cells showing spontaneous firing at rest, action potentials occurring outside the range of the current injection period were not included for induced action potential analysis. For Na^+^ currents analysis, leak current subtraction was performed in ClampFit to isolate depolarization steps induced inward Na^+^ currents; the peak amplitude of each step was used to plot the I‐V curve, and the maximum of the peak amplitude was used to calculate the current density. The outward currents induced by depolarizing steps near the end of voltage steps were used for the K^+^ current I‐V plot, and the currents at the +40 mV step were used to calculate the K^+^ current density. The experimenter was blinded to conditions during analysis.

### 2.5 Microfluidic Chambers

Methods of chamber preparation and use were based off previous publications [13, 14]. 24 × 30 rectangle glass coverslips were first exposed to a flame following submersion in 90% ethanol. They were then soaked in 70% ethanol for an hour with constant agitation, dried in the laminar flow hood, and then exposed to UV light for 30 min on each side. Sterilized coverslips were coated for differentiation as above with PLO and fibronectin. Silicone microfluidic devices with a 450 μm microgroove barrier (SND450; Xona) were soaked in 70% ethanol for an hour with constant agitation, dried in the laminar flow hood, and then exposed to UV light for 30 min on each side. Sterilized microfluidic devices were then adhered to coated coverslips with gentle pressure. On D0, 40 000 HD10.6 cells were seeded into the soma compartment in 20 μL proliferation media and incubated at 37°C and 5% CO_2_ for 10 min, prior to topping off media in the soma compartment. The following day, media within the soma compartment was changed to differentiation media and differentiation media was added with a 2X NGF concentration to the axon‐only compartment. Half media changes were performed every 2 days for each compartment.

### 2.6 AAV9‐mCherry Production

AAV was produced in house by triple‐transfection of HEK293T cells (RRID:CVCL_0063) and purified with an iodixanol gradient as previously described [15]. 56 h prior to transfection, cells were seeded at 10% confluency to achieve 80%–90% confluency at transfection. Media was exchanged completely 2 h pre‐transfection and 16 h post‐transfection. 15 cm plates were transfected with 30 μg total of transgene vector (mCherry), capsid vector (pXR‐9), and pHelper vector (Catalog No. V005569; NovoPro) in an equimass ratio utilizing linear polyethylenimine (PEI) at a ratio of 4:1 PEI to DNA (PEI dissolved 1 mg/mL in DPBS, pH balanced to 7 with HCl and NaOH). OptiMEM (ThermoFisher) was used to promote complex formation at a final volume of 500 μL transfection mixture per plate. Mixture was briefly vortexed then incubated at room temperature for 10 min before dropwise addition to cells. 84 h after transfection, virus was harvested from supernatant via overnight 4°C 10% polyethylene glycol (PEG) incubation and directly from freeze–thaw lysed cells. Benzonase (SigmaAldrich) incubation at 37°C for 1 h was used to digest any unencapsulated DNA. An iodixanol gradient was used to isolate virus which was then dialyzed using 50 kDa MWCO centrifugal filters (Millipore) in a solution of DPBS (ThermoFisher), 50 mM NaCl, and 0.0001% of Pluronic F68 (ThermoFisher) and concentrated to a final volume of ~200 μL. Viral titers were quantified via qPCR of serial dilutions using a known standard (VR‐1616; ATCC) and primers targeting the ITR regions: AAV‐ITR‐F (5′‐CGGCCTCAGTGAGCGA‐3′) and AAV‐ITR‐R (5′‐GGAACCCCTAGTGATGGAGTT‐3′).

### 2.7 AAV9‐mCherry Transduction

Within individual wells of a 24‐well plate, 1E8, 1E9, or 1E10 vg (viral genomes) of AAV9‐mCherry were added in 200 μL of differentiation media without doxycycline and incubated for 2 h at 37°C and 5% CO_2_, following which 400 μL of media with doxycycline was added to reservoirs. Within microfluidic chambers, 1E9 vg were added in 5 μL of differentiation media without doxycycline into the axon‐only compartment and incubated for 2 h at 37°C and 5% CO_2_, followed by the addition of media with doxycycline to reservoirs. A 100 μL volume differential was always maintained between axon and soma compartments.

### 2.8 Statistical Analyses

Results were analyzed using GraphPad Prism v.10 software (RRID:SCR_002798; GraphPad, San Diego, USA). Power analysis was used to determine the appropriate sample sizes for statistical analysis. The normal distribution of values in each analysis was performed with the Kolmorogov‐Smirnov test according to the sample size. When a non‐parametric statistical test was performed, the Mann–Whitney test was used to compare two means. When the comparison involved more than two means, the Kruskal‐Wallis test was performed to determine if there were differences between the groups analyzed. If the values followed a normal distribution, parametric statistical tests were performed. The *T*‐student test was selected when two means were compared. When the comparison involved more than two means, a one‐way or two‐way analysis of variance (ANOVA) was performed according to the experimental design followed. The level of significance adopted in each analysis of the results was *p* < 0.05. Specific analyses are included in the figure legends.

## 3 Results

### 3.1 Key Ion Channel Expression, Localization, and Function

**FIGURE 1:**
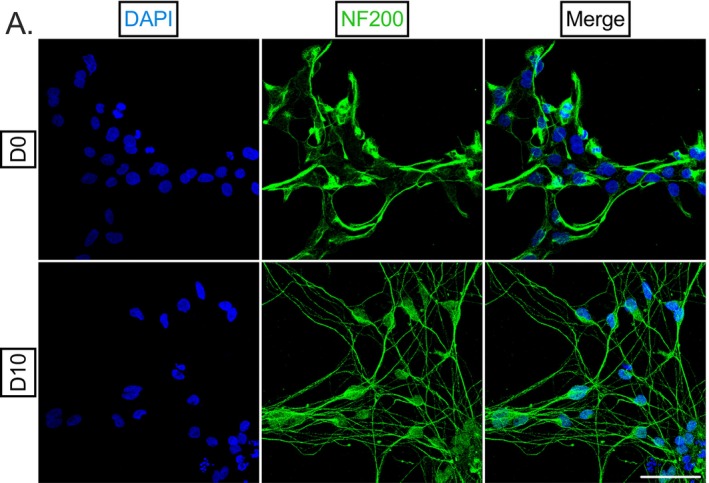
HD10.6 neuronal morphology. (A) Undifferentiated (D0) and differentiated (10 days post‐differentiation, D10) HD10.6 cells were fixed and stained for nuclear stain DAPI (blue) and neurofilament NF200 (green). Scale bar 50 μm. Representative images shown from three independent experiments.

To confirm the neuronal origin and nature of HD10.6 cells, we stained proliferating, undifferentiated HD10.6 cells (day 0, D0) and differentiated (10 days‐post differentiation, D10) HD10.6 cells for canonical markers of neurons (Figure 1). Sensory [16, 17] neuron neurofilament NF200 was detected abundantly and specifically within the cytoplasm of all D0/D10 HD10.6 cells (100%, Figure 1A) as identified by the presence of DAPI‐positive staining. Therefore, we proceeded to use NF200 as a positive control for staining in D0 and D10 cells. The presence of neuronal markers in D0 cells despite their relative immaturity is not surprising; immunoreactivity for neuronal microtubule component β3‐tubulin [10, 11] has previously been demonstrated in both D0 and D10 cells and likely reflects the neuronal lineage‐commitment of the HD10.6 cell line. Whereas the fate of stem cells is largely promiscuous or prone to change with any minor adjustment to differentiation media composition [18], HD10.6 cells retain their neuronal phenotype even when cultured in media that support the differentiation of cells into Schwann cells or smooth muscle cells [10]. We also confirmed the presence of pan‐neuronal markers β3‐tubulin and PGP9.5 within D0/D10 HD10.6 cells (data not shown). The structural nature of NF200 highlights the morphology of undifferentiated and differentiated HD10.6 cells. While D0 HD10.6 cells exhibit a fibroblast‐like morphology, rounded soma and axonal projections were readily visible by D4 post‐differentiation and became more distinct over time.

**FIGURE 2:**
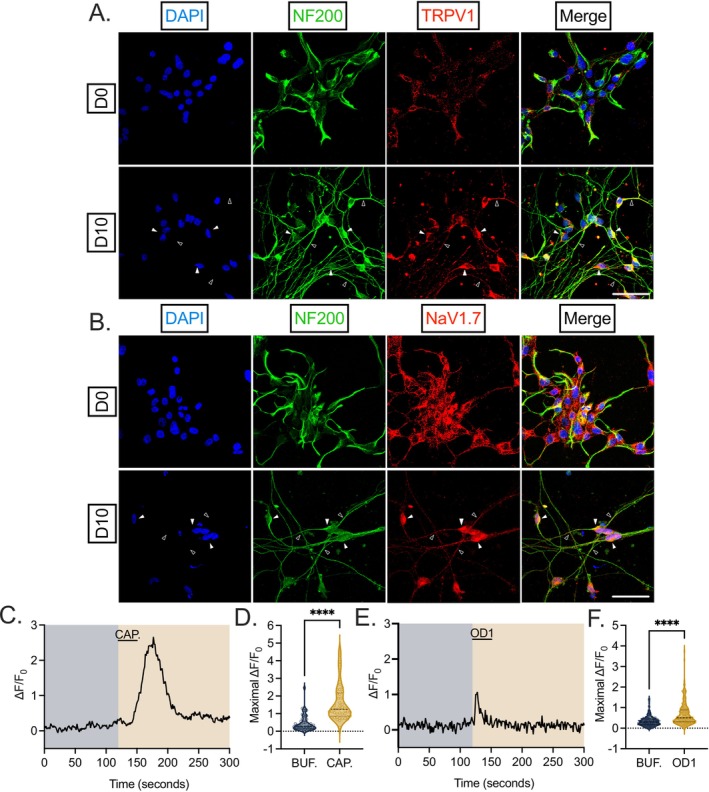
Key nociceptive ion channel abundance, localization, and activity in HD10.6 cells. (A, B) D0 and D10 HD10.6 cells were fixed and stained for DAPI (blue), NF200 (green), and nociceptive ion channel TRPV1 (A) or NaV1.7 (B) in red. Representative images shown from three independent experiments. Scale bar 50 μm; filled arrows indicate examples of signal at soma whereas empty arrows indicate signal within axons. (C–F) Calcium influx in response to buffer only (BUF., blue) as well as during and following 10 μM TRPV1 agonist capsaicin (CAP., C, D, orange) or 1 μM NaV1.7 agonist OD1 (E, F, orange) was recorded from differentiated HD10.6 cells with Ca^2+^ indicator Fluo‐4 AM (5 μM). Buffer was applied for 2 min and followed by the application of capsaicin or OD1 for 30 s. After up to 4 additional minutes of buffer, 50 mM KCl was applied for 5 s at the termination of the experiment to verify cell viability. Representative traces in response to respective stimuli shown (C,E), as well as the maximal normalized intensity (D,F). Replicates from three independent experiments. Statistical comparisons were made using Wilcoxon signed‐rank test due to non‐normality. *p < 0.05; **p < 0.01; ***p < 0.001; ****p < 0.0001.

The electrical excitability of nociceptors is partially regulated by ion channels, the activation of which enables neuronal depolarization, action potential generation, and the communication of stimuli. Select ion channels within nociceptors have been implicated in the initiation or persistence of pain states, and therefore their expression and function in this system is essential to investigate (Figure 2). As an example, non‐selective cation channel TRPV1 plays a crucial role in pain and nociceptive temperature sensation and can be activated by an array of stimuli, including capsaicin, heat, and low pH. Within D0 and D10 HD10.6 cells, we detected immunoreactivity for TRPV1, with localization of TRPV1 to both the soma (filled arrows) and axons (empty arrows) of differentiated HD10.6 cells (Figure 2A). No signal was detected in control slides incubated without primary antibodies. There are nine isoforms of voltage‐gated sodium channels (NaV1.1–1.9), and four isoforms expressed within the DRG (NaV1.3, NaV1.7–1.9) are implicated in the transmission of pain signals by epidemiological patient data and animal models [19]. Over the last few years, special attention has been paid to NaV1.7 for its potential therapeutic utility in small molecule or gene therapy form for a variety of chronic pain states [20, 21, 22, 23]. Therefore, we also investigated the expression of NaV1.7 within the HD10.6 cells and found that NaV1.7 was abundantly expressed along the soma and the axon in all differentiated HD10.6 cells (Figure 2B). No signal was detected in control slides incubated without primary antibodies. NaV1.7 protein has previously been observed in HD10.6 cells differentiated under a different protocol [12].

To confirm the electrical functionality of these characteristic ion channels, calcium influx events were tracked through live imaging following exposure to appropriate agonists. Acute (30s) exposure of HD10.6 cells to 10 μM TRPV1 agonist capsaicin induced a transient and statistically significant increase in calcium influx representative of membrane depolarization (Figure 2C,D), in agreement with a previous report [10]. We also probed the specific activity of NaV1.7 through calcium imaging with NaV1.7 agonist OD1. Scorpion toxin OD1 enhances NaV1.7 activity by inhibiting channel inactivation and increasing peak current [24, 25]. Intraplanar injection of OD1 into mice elicits spontaneous pain behavior [26]. In response to OD1 exposure, we detected a transient and significant increase in Ca^2+^ influx in differentiated HD10.6 cells (Figure 2E,F). The differential morphology displayed by calcium influx traces initiated by the addition of capsaicin and OD1 may reflect the distinct molecular mechanisms of these drugs performing as a channel agonist for TRPV1 and inactivation inhibitor for NaV1.7, respectively. Capsaicin directly activates TRPV1, a non‐selective cation channel with relatively slow activation and desensitization kinetics, resulting in a gradual increase in intracellular calcium followed by a prolonged plateau as calcium continues to enter through the open channel. In contrast, OD1 acts as an inactivation inhibitor of the voltage‐gated sodium channel NaV1.7, promoting persistent sodium influx and rapid membrane depolarization. Because the time scale of this altered inactivation is in the milliseconds to seconds range, this transient depolarization activates voltage‐gated calcium channels, producing a sharp and short‐lived calcium transient that rapidly decays as sodium channels eventually inactivate and membrane potential repolarizes. These distinct temporal profiles of the calcium signals are consistent with the fundamentally different biophysical properties and signaling mechanisms of TRPV1 and NaV1.7. Therefore, differentiated HD10.6 cells abundantly express characteristic nociceptive ion channels implicated in pain states, and these channels are responsive to direct stimulation.

### 3.2 Electrophysiological Properties of HD10.6 Cells

**FIGURE 3:**
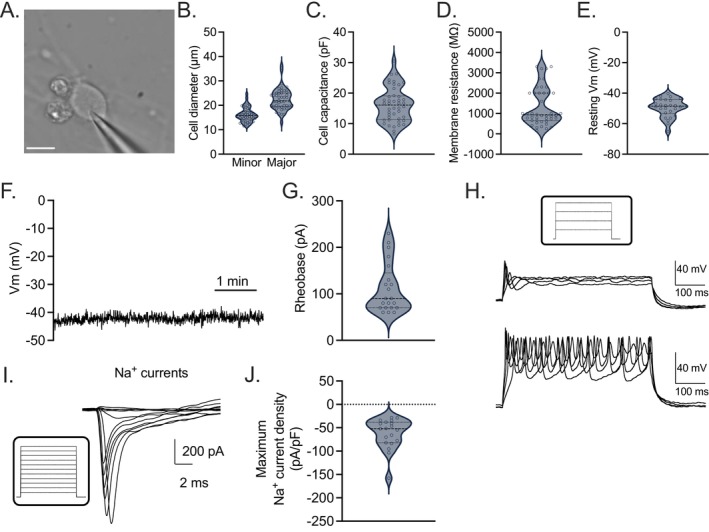
Electrophysiological parameters of differentiated HD10.6 cells. D10 HD10.6 cells under current clamp (A–H). (A) An image of differentiated HD10.6 undergoing active whole‐cell patch clamp. Scale bar 10 μm. (B) The minor and major diameters of cell soma. (C) Cell capacitance. (D) Membrane resistance. (E) Resting membrane potential. (F) Representative recording of stable resting membrane potential over time. (G) Rheobase. (H) Representative recording of single discharge (upper) and multiple discharge (lower) patterns following current injection. Inset: I‐command, 50 pA increment, 500 ms duration. D10 HD10.6 cells under voltage clamp (I–J). (I) Sodium currents elicited by depolarizations from a holding potential of −80 mV to test potentials from −70 mV to +40 mV with 10 mV increment (V‐command showing in the inset). (J) Maximum Na^+^ sodium current density. Replicates from three independent experiments.

We next performed whole‐cell patch clamp electrophysiology, including current clamp and voltage clamp, on differentiated D10 HD10.6 cells (Figure 3A) to outline their electrophysiological profile. Due to the oval shape of the cells, the diameters of both the major axis and minor axis were recorded. The average minor and major diameters of the HD10.6 cell soma measured 16.2 ± 0.4 μm and 22.5 ± 0.6 μm, respectively (Figure 3B). The mean capacitance was 19.3 ± 0.9 pF (Figure 3C), and the mean membrane resistance was 1328 ± 140 MΩ (Figure 3D). Under current clamp, the resting membrane potential was valued at −49.5 ± 1.2 mV (Figure 3E) and remained stable during the recording of untreated HD10.6 cells (Figure 3F). Actional potential discharges were detected following current injection, and the average rheobase of differentiated HD10.6 cells was 111 ± 12 pA (Figure 3G). Most differentiated HD10.6 cells investigated (92%) released a single discharge in current step recordings (Figure 3H, top). However, a smaller proportion (8%) emitted two or more sequential discharges (Figure 3H, bottom). Under voltage clamp, we identified sodium currents elicited by depolarizations (Figure 3I), confirming the activity of sodium channels. The sodium current density averaged 62.5 pA/pF (Figure 3J).

### 3.3 Afferent Peptide Expression and Localization

**FIGURE 4:**
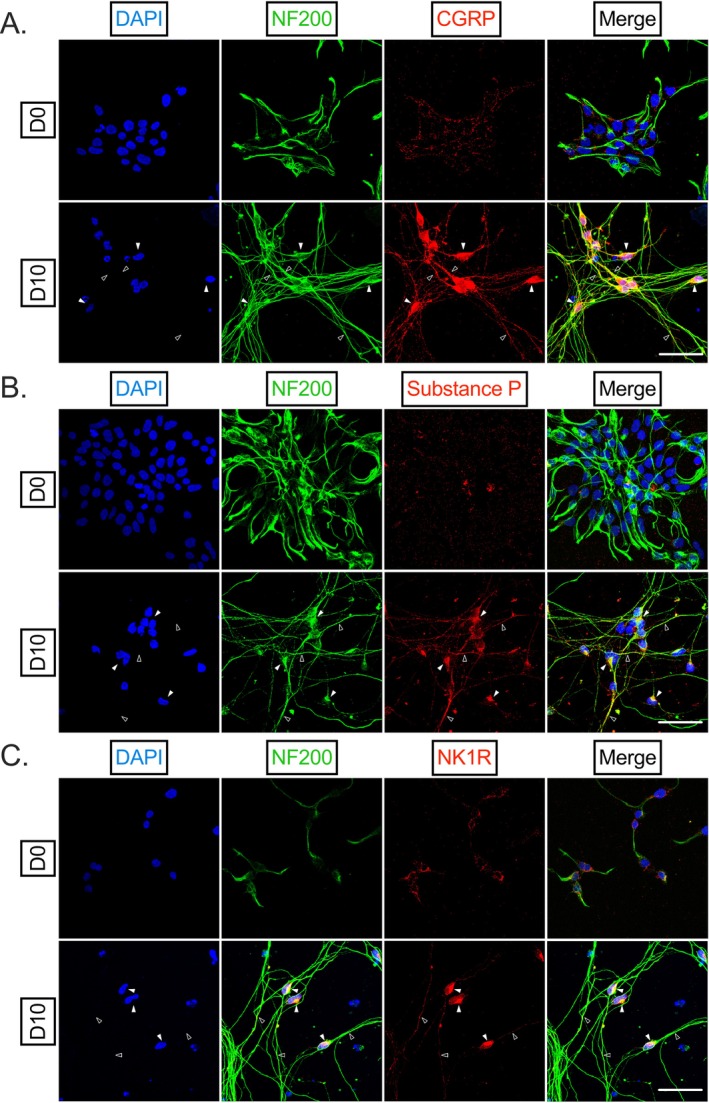
HD10.6 cells express key afferent peptides and related machinery associated with pain regulation. (A–B) D0 and D10 HD10.6 cells were fixed and stained for DAPI (blue), NF200 (green), and afferent peptides CGRP (A) or Substance P (B), or Substance P receptor NK1R (C) in red. Representative images shown from three independent experiments. Scale bar 50 μm; filled arrows indicate examples of signal at soma whereas empty arrows indicate signal within axons.

The peripheral terminal of the nociceptor acts not only as a sensor of local (painful) stimuli, but also as an effector [27]. Nociceptors can store and release afferent peptides, such as CGRP and substance P, which can have excitatory impacts on non‐neuronal cells of the DRG through neurogenic inflammation and may directly feedback upon the nociceptor itself [28]. We therefore inquired whether HD10.6 cells expressed, at the protein level, characteristic afferent peptides (Figure 4). Indeed, we detected faint CGRP (Figure 4A) and substance P (Figure 4B) within undifferentiated HD10.6 cells, and this signal grew more abundant following differentiation. The receptor for SP, neurokinin 1 receptor (NK1R) [29], is expressed by vascular endothelial cells and immune cells, which contribute to the inflammatory aftermath of SP release. However, there is also evidence that nociceptors express NK1R on their surface [30], suggesting the existence of an excitatory loop within the DRG wherein afferent peptides may feedback on the nociceptor. We inquired into the expression of NK1R within HD10.6 cells and found abundant NK1R staining in undifferentiated and differentiated HD10.6 cells (Figure 4C). No signal was detected in control slides incubated without primary antibodies in any of these experiments. Therefore, differentiated HD10.6 cells abundantly express excitatory afferent peptides, as well as the substance P receptor. Importantly, these data suggest that HD10.6 cells can be used to study the neurogenic contributions from and to human nociceptors.

### 3.4 Functional Opioid‐Induced Analgesic Signaling Within HD10.6 Cells

**FIGURE 5:**
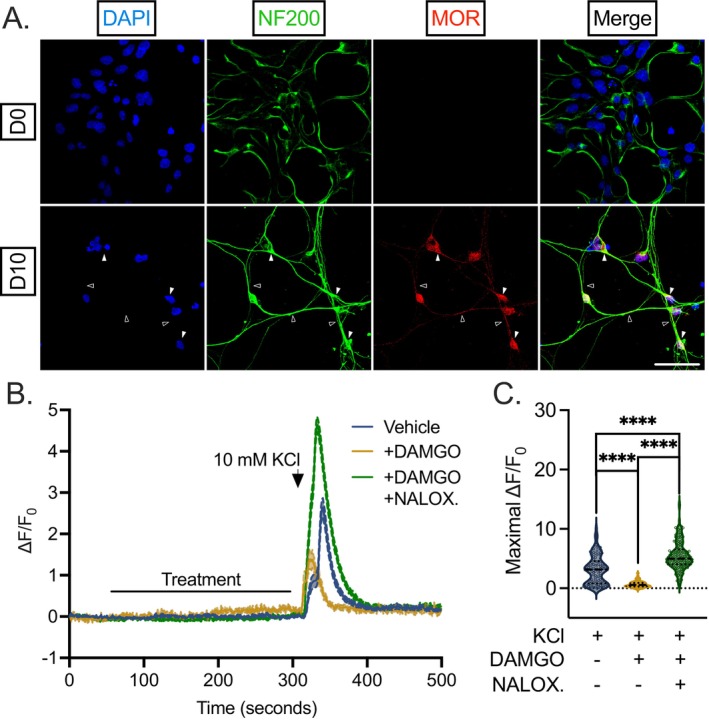
Functional opioid‐induced analgesic signaling in human HD10.6 cells. (A) D0 and D10 HD10.6 cells were fixed and stained for DAPI (blue), NF200 (green), and mu opioid receptor 1 (MOR1, red). Scale bar 50 μm; filled arrows indicate examples of signal at soma whereas empty arrows indicate signal within axons. (B–C) Calcium influx in response to 10 mM KCl was recorded from differentiated HD10.6 cells with Fluo‐4 AM. Buffer was applied for 1 min. Following an additional 4‐min perfusion of buffer with vehicle (blue), 1 μM DAMGO (orange), or both DAMGO and naloxone (NALOX.) at final concentrations of 1 μM and 10 μM, respectively (green), a 10‐s application of 10 mM KCl was applied. After up to 4 additional minutes of buffer, 50 mM KCl was applied for 5 s at the termination of the experiment to verify cell viability. Combined traces (mean ± SEM, B), as well as maximal normalized intensity (C) in response to respective treatments. Replicates from three independent experiments. Statistical comparisons were made using an unpaired non‐normal *t*‐test (Mann–Whitney *U* test). *p < 0.05; **p < 0.01; ***p < 0.001; ****p < 0.0001.

Opioid signaling has long been co‐opted for significant pain relief in humans, although with substantial issues regarding dependency and addiction [31]. The role of opioid signaling within the peripheral terminal, in addition to the spinal cord, has become increasingly appreciated [32]. Rodent model neurons do not recapitulate the diversity and robustness of mu opioid receptors (MOR) within nociceptors [8], nor do they entirely accurately recapitulate opioid use disorder [33] at the transcriptional and epigenetic level, emphasizing the importance of human material in this study. We therefore inquired whether functional mu opioid signaling was present in differentiated HD10.6 cells (Figure 5). First, we stained both D0 and D10 HD10.6 cells for the mu opioid receptor (MOR). We found lowly detectable signal in D0 HD10.6 cells, but more abundant signal within the soma and axons of differentiated HD10.6 cells (Figure 5A). To examine the functional capacity of opioid receptors to modulate membrane depolarization, electrical excitation was initiated with 10 mM KCl and measured by calcium influx in the absence or presence of pre‐treatment with the mu opioid selective agonist DAMGO. Pre‐treatment with DAMGO for 2 min resulted in a strikingly attenuated calcium influx peak in response to KCl treatment (Figure 5B), and the change in maximal calcium influx intensity was reduced by approximately 50% (Figure 5C). This reduction in influx was not due to cell death, as treated cells responded to 50 mM KCl at the termination of the experiment. Importantly, the effect on influx by DAMGO was also reversed through the addition of opioid receptor antagonist naloxone, which enhanced influx over control conditions as determined by maximal intensity (25% increase, Figure 5C). In agreement with previous reports [34], this potentiation following naloxone addition suggests the existence of opioid signaling in the absence of agonist binding, which is itself inhibited by naloxone through an inverse agonist effect [35, 36, 37, 38]. The addition of DAMGO or the DAMGO/naloxone combination alone (in the absence of 10 mM KCl) did not induce calcium influx levels greater than those observed during perfusion with buffer. Therefore, differentiated HD10.6 cells display functional mu opioid signaling and can be used to model these phenomena.

### 3.5 Peripheral Sensitization: An Inflammatory Soup Elicits and Primes Nociceptive Signaling in HD10.6 Cells

**FIGURE 6:**
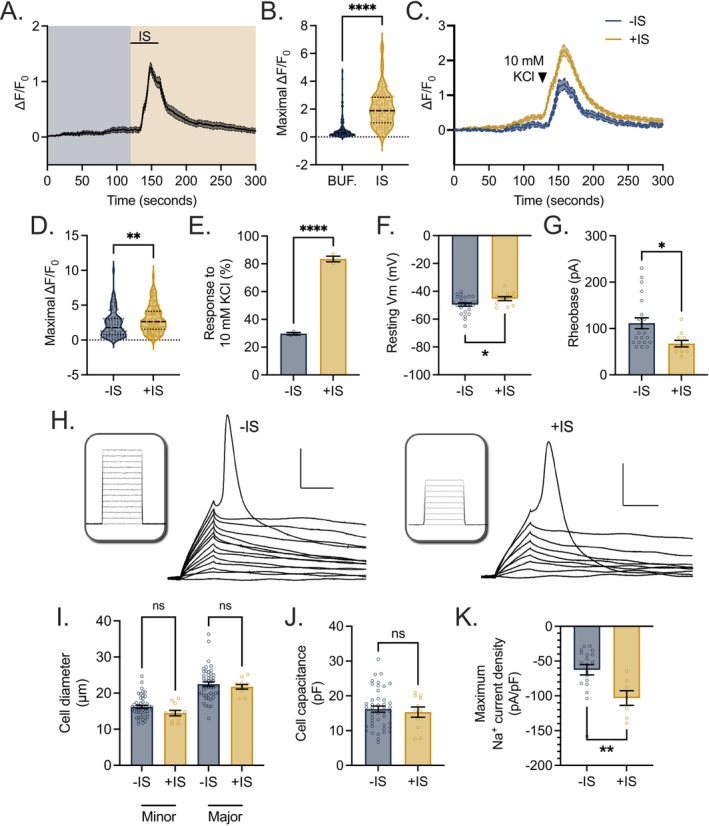
HD10.6 cells undergo peripheral sensitization in response to an inflammatory cocktail. (A, B) Calcium influx in response to buffer only (BUF., blue) as well as during and following application of inflammatory soup (IS, orange) was recorded from differentiated HD10.6 cells with Fluo‐4 AM. Buffer was applied for 2 min, and IS was applied for 30 s. After up to 4 additional minutes of buffer, 50 mM KCl was applied for 5 s at the termination of the experiment to verify cell viability. Combined traces (mean ± SEM) in response to stimuli shown (A), as well as maximal normalized intensity (B). Replicates from two independent experiments. (C–K) D10 HD10.6 cells were incubated with vehicle (−IS) or IS for 4 h. (C–E) Following incubation and thorough washing, calcium influx in response to buffer and a 10‐s application of 10 mM KCl was recorded. After up to 4 additional minutes of buffer, 50 mM KCl was applied for 5 s at the termination of the experiment to verify cell viability. Combined traces (mean ± SEM) in response to treatment and stimuli shown (C), as well as maximal normalized intensity (D) and the percentage of HD10.6 cells responsive to 10 mM KCl (E). Replicates from two independent experiments. (F–J) Following incubation and thorough washing, whole‐cell patch clamp was performed on HD10.6 cells. (F) Resting membrane potential. (G) Rheobase. (H) Representative rheobase recordings. Insets: I‐command, 10 pA increment, 10 ms duration. Scale bar: 20 mV, 10 ms. (I) The minor and major diameters of cell soma. (J) Cell capacitance. (K) Following incubation and thorough washing, voltage clamp recordings were performed on HD10.6 cells. Maximum Na^+^ current density is shown. Statistical comparisons were made using an unpaired *t*‐test or Wilcoxon signed‐rank test dependent on normality of the data. *p < 0.05; **p < 0.01; ***p < 0.001; ****p < 0.0001.

The above data suggest that HD10.6 cells act as *de facto* human nociceptors and respond electrically to direct agonists and antagonists of ion channels which mediate excitability. We next sought to perturb the excitability of the differentiated HD10.6 cells in a physiological manner that mirrors a painful condition to which a nociceptor might be subject (Figure 6). We therefore exposed HD10.6 cells to an “inflammatory soup” (IS) [39, 40, 41], a mixture including bradykinin, prostaglandin E2 (PGE2), serotonin, adenosine triphosphate (ATP), and histamine, molecules released at the site of injury/inflammation as a result of the disruption of surrounding inflammatory cells. We aimed to model human “pain” using nociceptor excitability/activity as the best readout second only to behavior. First, we performed calcium influx live‐imaging on the HD10.6 cells in response to transient (30s) IS application to assess whether this cocktail could directly induce excitation of the HD10.6 cells. In the scenario of acute pain, inflammatory mediators induce excitation and a pain state that subsequently resolves [42]. IS addition almost instantly induced a statistically significant and transient influx in calcium mirroring membrane depolarization (Figure 6A,B). Next, we queried whether transient IS application would prime the HD10.6 cells analogous to a process undergone by nociceptors known as peripheral sensitization [43]. This process, also known as hyperalgesic priming, is commonly used to study how acute pain transforms into chronic pain [44]. HD10.6 cells were pre‐incubated with IS or vehicle control for a 4‐h period, following which the HD10.6 cells were thoroughly washed and prepared for calcium imaging where 10 mM KCl was used to initiate electrical excitation. HD10.6 cells pre‐treated with IS responded to 10 mM KCl significantly more robustly than vehicle controls in terms of the fold change/potency of calcium influx within HD10.6 cells (Figure 6C,D) as well as the percentage of HD10.6 cells that were responsive to the stimulus (Figure 6E). Excitingly, these data suggest that the IS can be applied to HD10.6 cells to model nociceptive signaling in both the presence and following the removal/resolution of a noxious stimulus. We verified this finding using the gold standard of membrane physiology, whole‐cell patch clamp. Differentiated HD10.6 cells pre‐incubated with IS for 4 h had a more depolarized resting Vm compared to untreated cells (Figure 6F, −IS: −49.5 ± 1.2 mV, *n* = 26; +IS: −45.3 ± 1.5 mV, *n* = 11; unpaired *t*‐test, *p* < 0.05). The rheobase was also significantly lower for +IS conditions than that observed for untreated cells (Figure 6G,H, −IS: 111 ± 12 pA, *n* = 21; +IS: 67 ± 7 pA, *n* = 11; unpaired *t*‐test, *p* < 0.05), and 30% of IS‐treated cells showed multiple discharges in current step recordings. These data indicate that HD10.6 cells incubated with IS are more likely to fire action potentials than untreated cells. No difference in cell capacitance or the minor/major diameters of cell soma between the IS‐treated and untreated cells was observed (Figure 6I,J), which suggests that IS incubation did not induce significant cell swelling, a common indicator of compromised cellular health. Under voltage clamp, a more than 2‐fold increase in sodium current density was identified in IS‐treated versus control‐treated differentiated HD10.6 cells (Figure 6K). This indicates that IS treatment markedly enhances functional sodium channel availability and/or activity at the membrane, consistent with an increase in intrinsic neuronal excitability. Such changes in sodium current density could lower the action potential threshold and facilitate repetitive firing, providing a mechanistic basis for the heightened excitability observed under current‐clamp conditions. Importantly, we conclude that HD10.6 cells undergo peripheral sensitization and can therefore be used to model both acute and persistent pain states.

### 3.6 The Peripheral Terminals of HD10.6 Cells Show AAV Uptake, Transport, and Soma Transfection

**FIGURE 7:**
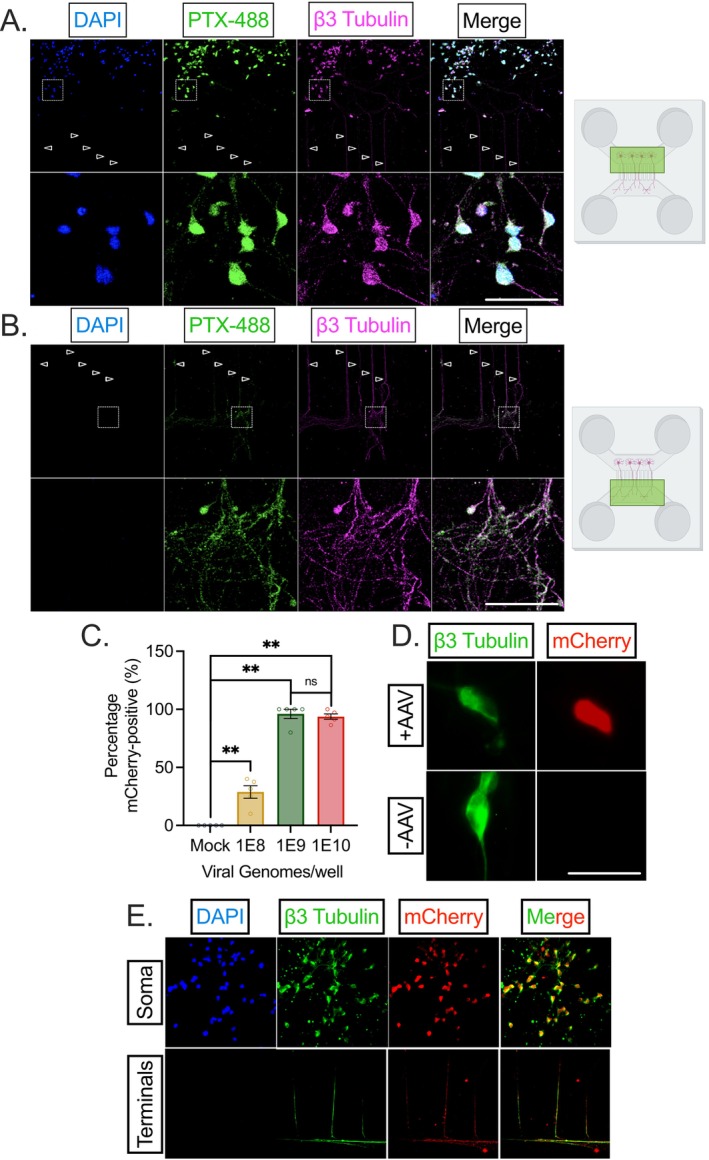
Culture of HD10.6 cells into microfluidic devices to study peripheral administration. (A, B) D0 HD10.6 cells were seeded into the soma compartment of a microfluidic chamber, and media was changed to differentiation media 24 h post‐seeding. A 2X nerve growth factor (NGF) gradient was used to guide axons towards the axon‐only compartment. At D14, the cultures within the chamber were fixed and stained for neuronal marker β3 tubulin (magenta), marker for NaV1.7 PTX‐488 (green), and DAPI (blue). Empty arrows point to axons traveling through microgrooves. (A) captures a representative image of the soma compartment and axons within the proximal end of the microgrooves, whereas (B) shows the axon‐only compartment and distal microgrooves. Scale bar 50 μm. Respective compartments are highlighted in green within the right‐hand animated microfluidic chambers; images of microfluidic chambers created with BioRender.com. (C, D) D10 HD10.6 cells in 24‐well cell culture wells were infected with AAV9‐mCherry. 3 days following infection, cells were fixed and stained for β3 tubulin (green), mCherry (red), and DAPI (blue) The percentage of mCherry+ HD10.6 cells based on the total number of cells identified by DAPI and β3 tubulin‐positive staining is demonstrated in (C). A representative image of AAV9‐mCherry‐treated (1E9, top) and untreated (bottom) HD10.6 cells at this time‐point is shown in (D). (E) 3 days following AAV9‐mCherry infection in the peripheral compartment of HD10.6 cells cultured in microfluidic chambers, the cultures were fixed and stained for β3 tubulin (green), mCherry (red), and DAPI (blue). Representative image of soma (top) and peripheral terminal compartments (bottom) demonstrate mCherry signal within the soma and axons of HD10.6 cells. Replicates from three independent experiments; statistical comparisons were made using an unpaired non‐normal *t*‐test. *p < 0.05; **p < 0.01; ***p < 0.001; ****p < 0.0001.

One of the limitations of many current in vitro nociceptive models is the disorganization of these cultured primary or immortalized DRG neurons within wells. Regarding the primary afferent, the nociceptor soma resides in the DRG and the peripheral axon terminals, which detect and respond to painful stimuli innervate the periphery a distance away. These are distinct physiological environments in that the soma and peripheral axons are in contact with differential local/circulating inflammatory products and non‐neuronal cells. The intrinsic biology of the soma and axon of any neuron is also distinct in terms of signaling integration, action potential initiation, and the distribution and influence of ion channels [45]. We therefore optimized the culture of HD10.6 cells into microfluidic chambers in which we can fluidically isolate, stimulate, and study the peripheral terminal and soma to effectively mirror the anatomy of a human patient (Figure 7).

Within the microfluidic device, there are separate soma and axonal compartments which are connected by 2 μm microgrooves. These microgrooves are large enough for the passage of neuronal axons but not cell bodies. D0 HD10.6 cells were seeded into the soma compartment of the microfluidic chamber and differentiated 24 h later. A nerve growth factor (NGF) gradient was used to guide axons from the soma compartment through microfluidic grooves towards the axon‐only compartment. By 5–8 days post‐differentiation, axonal extensions sprouted into the axon‐only compartment. The length of the microgrooves in this system is 450 μm, therefore reflecting the tremendous capability of HD10.6 cells to sprout. At D15, the cultures within the chamber were fixed and stained for neuronal marker β3‐tubulin, marker for NaV1.7 ATTO488‐ProTx‐II (PTX‐488) [46], and DAPI. Within the soma compartment, we demonstrate the confinement of viable cell bodies through the presence of rounded soma staining positive for both PTX‐488 and DAPI (Figure 7A). Robust dendritic branching is apparent through the microgrooves (empty arrowheads) and in the axon‐only compartment representing the peripheral terminal (Figure 7B).

This anatomical distinction is advantageous to study not only the physiology of pain states, but also its therapy. The use of gene therapies such as adeno‐associated virus (AAV) and antisense oligonucleotides (ASO) [47] to treat chronic pain states is a rapidly advancing field. While these vectors are commonly delivered through an intrathecal injection (analogous to treatment in the soma compartment of the HD10.6 cells within the microfluidic chamber), their peripheral administration is being increasingly investigated and applied [48, 49] due to efficacy, convenience, and anatomic specificity. Peripheral administration of the therapy, such as through subcutaneous injection, occurs at the periphery from where the AAV/ASO must infect the peripheral terminal endings and undergo retrograde transport to travel to the soma of the nociceptor. We recognized the suitability of the system of HD10.6 cells cultured within the microfluidic chambers to examine whether infection within the peripheral terminal would be a plausible route of inoculation in human material. Following the optimization of viral titer for mCherry‐AAV9 uptake (Figure 7C,D), the peripheral terminal was infected with 1E9 vg [50]. Three days later, chambers were fixed and stained for mCherry. mCherry staining, detected in both the soma and axons, was present in 100% of HD10.6 cells as identified by the presence of β3‐tubulin [10, 11] (Figure 7E). Therefore, AAV9 infection, retrograde transport, and expression proceed within HD10.6 cells cultured in microfluidic chambers, having wide‐ranging implications for future studies on not just mechanisms of pain signaling and chronification, but also its therapy.

## 4 Discussion

Since the introductory publication of HD10.6 cells, it has not been concluded whether these neuronal cells are in fact nociceptors versus alternative DRG neuron subtypes [10], and few other groups have employed or characterized these cells [11, 12, 51, 52]. We systematically characterized the presence of nociceptive machinery within the neuronal soma and axons at the protein level, including TRPV1, NaV1.7, CGRP, Substance P, and NK1R. We verified through calcium influx assays that differentiated HD10.6 cells respond to direct agonists of TRPV1 and NaV1.7 and characterized the electrophysiological profile of differentiated HD10.6 cells through whole‐cell patch clamp. We found that mu opioid administration robustly diminishes electrical activity, and that this phenomenon can be recovered with the addition of naloxone, demonstrating the existence of functional mu opioid signaling as observed in human patients. Importantly, we also found that HD10.6 cell excitability can be enhanced through the addition of an inflammatory soup, leading to both the direct activation of excitable events, as well as the peripheral sensitization process that is essential to developing persistent pain states. Finally, we optimized the culture of HD10.6 cells into microfluidic chambers to enable the physiological investigation of the soma versus the peripheral terminal. We found that HD10.6 cells successfully take up AAV through infection of the peripheral terminal, mirroring peripheral therapeutic administration. Altogether, we have developed the use of HD10.6 cells into a model of human nociception in a dish that can be employed for the investigation of molecular mechanisms of pain signaling and its therapy.

To address the physiological relevance and limitations of this system, the electrophysiological parameters of differentiated HD10.6 cells should be compared to those of human patient DRG neurons matured in vivo (human donor cells, HDCs hereafter), as well as alternative experimental systems of human nociceptor‐like neurons created through induced pluripotent stem cell protocols (NLHIPSCs hereafter) [53, 54, 55]. HD10.6 cells differentiated under our current protocol share several electrophysiological parameters with HDCs. First, C‐fiber nociceptors are classified as “small diameter” DRG neurons [6, 56]; accordingly, the average minor and major diameters of the HD10.6 cell soma measure 16.2 ± 0.4 μm and 22.5 ± 0.6 μm respectively (Figure 3B). The diameter of NLHIPSCs averages 21.7 ± 0.4 μm [57] and the diameter of HDCs ranges from 20–90 μm [58]. For D10 HD10.6 cells, the mean capacitance was 19.3 ± 0.9 pF (Figure 3C), a value more closely resembling that of NLHIPSCs (23.1 ± 1.1 pF) than that of HDCs (104.0 ± 26.2 pF) [57, 58] likely due to the smaller soma diameter of the differentiated HD10.6 cells and NLHIPSCs. The membrane resistance of D10 HD10.6 cells (1328 ± 140 MΩ, Figure 3D) was higher than that of both HDCs and NLHIPSCs (248.2 ± 31.1 MΩ and 339.2 ± 12.6 MΩ respectively [57, 58]), indicating that HD10.6 cells may express less leak type K^+^ channels.

Under current clamp, differentiated HD10.6 cells exhibited a stable resting membrane potential during recording (Figure 3F), and therefore an absence of substantial ectopic activity that would otherwise nullify our ability to study the initiation of aberrant excitation and the associated pain state. Healthy DRGs isolated from human patients also demonstrate a stable resting membrane potential, whereas DRGs isolated from painful dermatomes display spontaneous ectopic activity [59, 60]. However, the value of resting membrane potential of differentiated HD10.6 cells was −49.5 ± 1.2 mV (Figure 3E), a value higher than that which has been reported for HDCs and NLHIPSCs with respective means and SEM of −54.9 ± 0.7 mV and −56 ± 0.9 mV, which were not statistically significant. A significant difference in rheobase was previously detected between HDCs and the NLHIPSCs (593 ± 63 vs. 74 ± 7 pA, respectively), *p* < 0.0001 [57]. The average rheobase of differentiated HD10.6 cells was 111 ± 12 pA (Figure 3G), more closely mirroring that of NLHIPSCs. The slightly higher resting membrane potential and lower rheobase of the HD10.6 cells in comparison to HDCs suggest that the HD10.6 system is not a faultless physiological recapitulation of healthy primary nociceptors. However, these data, with the report of a stable resting membrane potential in D10 HD10.6 cells, also suggest that the study of the initiation and/or chronification of pain in vitro may be easier to robustly and reproducibly achieve in the HD10.6 system in comparison to healthy primary nociceptors. Further, two additional factors should be considered when interpreting this depolarized resting Vm. First, we followed the methodology described in the original characterization of the HD10.6 cell line, which used an extracellular bath solution containing 4 mM potassium [10]. In contrast, most studies of human DRG neurons and iPSC‐derived sensory neurons use extracellular potassium concentrations closer to 3 mM. Based on the Goldman–Hodgkin–Katz (GHK) equation, and assuming comparable relative ionic permeabilities across the membrane of HD10.6 cells, primary human DRG neurons, and iPSC‐derived neurons, this difference in extracellular potassium concentration alone would be expected to depolarize the resting Vm by approximately 2–3 mV under our recording conditions. Second, the high membrane resistance of HD10.6 cells may contribute to an underestimation of the true resting membrane potential during whole‐cell current‐clamp recordings. When recording the resting Vm in current‐clamp mode (I = 0), the membrane resistance (Rm) and the seal resistance (Rseal) effectively form a voltage divider. Under these conditions, the recorded membrane potential is given by: Vm, recorded = Em × Rseal / (Rseal + Rm) where Em represents the true resting membrane potential [61, 62]. Assuming a typical seal resistance of ~2 GΩ and a membrane resistance of ~1 GΩ in HD10.6 cells, the recorded resting Vm could underestimate the true Em by approximately 33%.

Most differentiated HD10.6 cells investigated (92%) released a single discharge in current step recordings (Figure 3H, top). However, a smaller proportion (8%) emitted two or more sequential discharges (Figure 3H, bottom). Whether the cell emitted a single or multiple discharge(s) was independent of increasing injected current, reflecting an intrinsic commitment to the discharge pattern. This heterogeneity in discharge pattern also occurs in both NLHIPSC and HDC systems [57, 58], but is unexpected within HD10.6 cells and (some) NLHIPSCs due to the cells' monoclonal nature. The variability in discharge patterns persisted in our experiments throughout biological replicates and passage number, suggesting it is not a result of genetic instability due to repeated cell division over time. In support of this speculation, the original HD10.6 publication provided evidence of genomic stability over time [10]. The heterogeneity of repetitive action potential generation between cells may arise due to the location of individual cells within the culture, with cells near the perimeter of the coverslip potentially exhibiting more free nerve endings and less soma‐soma contact. The possibility of a subpopulation arising, however unlikely, cannot be excluded.

Under voltage clamp, we identified sodium currents elicited by depolarizations (Figure 3I), confirming the activity of sodium channels. The sodium current density averaged 62.5 pA/pF (Figure 3J), a value resembling that observed from HDCs (mean: 60.5 pA/pF) [63]. In conclusion, HD10.6 cells differentiated under our current described protocol share key electrophysiological properties with nociceptive neurons from human patients, including a small diameter, stable resting membrane potential, single and multiple discharge patterns, and sodium current density values.

Our HD10.6 differentiation protocol is modified from that of previous publications as it does not employ the common maturation supplement forskolin (FSK). In previous work, HD10.6 cells were cultured with up to 25 μM FSK [12, 51]. When HD10.6 cells were cultured with 25 μM FSK in addition to the differentiation media described in the Methods, the average resting membrane potential was 30% higher (approximately −35 mV) than that observed under incubation without FSK (data not shown). Action potentials were difficult to induce with current injections, suggesting the FSK concentration and/or incubation time (10 days) led to premature depolarization and the failure of action potential generation. The sodium current density averaged 62.5 pA/pF in HD10.6 cells differentiated under our FSK‐free protocol, a value closely resembling that observed from HDCs (mean: 60.5 pA/pF) [63]. In contrast, incubation under 25 μM FSK in our hands led to an average sodium current density of 152.7 pA/pF. It was previously reported that most cells under the 25 μM forskolin protocol demonstrated high sodium current densities, with 40% of cells exhibiting values over 80 pA/pF [12]. We therefore conclude that FSK addition in our hands, at the 25 μM concentration and/or 10‐day length of incubation time, results in a depolarized, non‐functional state. This depolarized state is possibly due to activation of cAMP signaling by FSK [64], which leads to PKA activation and further enhancement of NaV1.8 membrane trafficking, as suggested in rodent studies [65, 66, 67]. The protocol herein avoids this FSK‐induced artifact, making subsequent findings more reliable than previously published work.

Recently, an elegant human DRG organoid system was constructed following the single‐cell spatiotemporal analysis of human embryonic DRGs at the transcriptomic level [68]. This system recapitulates all cell types present in the DRG, including multiple neuronal subtypes, glia, and Schwann cells, but it also requires regular access to human embryonic DRGs as well as a 90 day‐differentiation period, leading to concerns over feasibility. The HD10.6 cell system does not carry all the components of a DRG, which is both a deficiency and benefit in that it provides a reductionist approach to studying the nociceptor. Nociceptor‐intrinsic signaling can be analyzed without complication, and the impact of individual additional cell types or immune components can be deciphered. In our studies, we use a physiological inflammatory cocktail containing products released under inflammation or injury that excite and sensitize the nociceptor. Under stimulus, PGE2 is released from immune cells, epithelial cells, and stromal cells; ATP from macrophages, neutrophils, and endothelial cells; and serotonin/histamine/bradykinin from mast cells and basophils. In this way, we account for the contributions of non‐nociceptor cells to pain sensation and signaling, and we can further dissect the individual contributions of each soup “ingredient” to altering HD10.6 excitability in future studies.

The experimentation concerning the addition of inflammatory soup (IS) provides insight into the mechanism of priming by inflammation mediators. The pre‐treatment of differentiated HD10.6 cells with IS led to a persistent and significant increase in resting membrane potential and sodium current density, as well as a decrease in rheobase (Figure 6). This data was accompanied by live calcium influx imaging data demonstrating an increase in the number of cells responsive to 10 mM KCl following IS pre‐treatment. The doubling of the sodium current density following IS treatment (Figure 6K) is directly indicative of greater expression of voltage‐gated sodium channels on the membrane. Increased trafficking of sodium channels, and specifically NaV1.7, upon inflammatory soup addition has previously been shown in primary rat and mouse DRG neurons [39, 40]. Our data now provides functional support for an inflammatory‐driven channel trafficking mechanism of peripheral sensitization within human nociceptors.

This finding of IS‐induced excitation and sensitization within the differentiated HD10.6 cells is also a key development in the nociceptive modeling of the HD10.6 cells. A sensitization phenotype is enormously difficult to achieve in nociceptor‐like human iPSCs despite optimization under various differentiation protocols, inflammatory soup compositions, and electrophysiological parameter evaluation ([69]). This sensitization potential, and the ability for extended culture (excellent cell viability following 30+ days post‐differentiation was noted, data not shown), enables the use of HD10.6 cells differentiated under this protocol for the study of chronic pain. To enhance the translatability of this system for chronic pain therapy, we cultured HD10.6 cells into a microfluidic chamber that mirrors the anatomy of the peripheral terminal and therapeutic administration. Locally targeted therapies, including topical ointments or genetic vectors through subcutaneous injection, can be administrated into the pseudo‐human periphery through incubation in the peripheral terminal axon end. In this work, we found that AAV9‐mCherry is successfully taken up in HD10.6 cells following infection of the peripheral terminal. Limited work has been performed following subcutaneous injection administration of AAV therapies [48, 49] despite its advantages [70, 71]. We therefore put forward this model as a general human screening system for the efficacy of both soma and axon/terminal administered therapeutics. We hope to further adapt this system to pathological pain states to more accurately mirror the peripheral terminal of a human patient.

## Author Contributions

**Sara A. Dochnal:** conceptualization, methodology, investigation, data curation, data analysis, writing (original draft). **Yixing Du:** methodology, investigation, data curation, data analysis, writing (review and editing). **Daniella Bandari:** investigation, data curation, data analysis, writing (review and editing). **Kaue Franco Malange:** data analysis, writing (review and editing). **Jack Bryant:** methodology, writing (review and editing). **Julia Borges Paes Lemes:** methodology, writing (review and editing). **Abby Whitford:** methodology. **Anna R. Cliffe:** methodology, resources, writing (review and editing). **Prashant Mali:** methodology, resources, writing (review and editing). **Kim Dore:** supervision, project administration, resources, writing (review and editing). **Yury I. Miller:** conceptualization, supervision, project administration, resources, writing (review and editing). **Tony L. Yaksh:** conceptualization, supervision, project administration, resources, writing (review and editing).

## Funding

This work was supported by HHS | NIH | National Institute of Neurological Disorders and Stroke (NINDS), NS132483, NS131560. HHS | NIH | National Institute on Aging (NIA), AG081037. HHS | NIH | National Heart, Lung, and Blood Institute (NHLBI), HL171505. HHS | NIH | National Institute of Diabetes and Digestive and Kidney Diseases (NIDDK), DK007044‐44. HHS | NIH | National Human Genome Research Institute (NHGRI), HG012351. HHS | NIH | National Institute of Allergy and Infectious Diseases (NIAID), AI185862. U.S. Department of Defense (DOD), W81XWH‐22‐1‐0401. HHS | NIH | National Institute of Neurological Disorders and Stroke (NINDS), NS047101.

## Conflicts of Interest

Mali reported that they are a scientific co‐founder of Shape Therapeutics, Navega Therapeutics, Pi Bio, Boundless Biosciences, and Engine Biosciences. Yaksh reported that he is a co‐founder of Raft Pharmaceutics and is a consultant for Navega Therapeutics. The terms of these respective arrangements have been reviewed and approved by the University of California San Diego in accordance with its conflict‐of‐interest policies.

## Data Availability

The data that support the findings of this study are openly available in Dryad through a unique, permanent digital object identifier: https://doi.org/10.5061/dryad.x95x69pz9.
